# The textual characteristics of traditional and Open Access scientific journals are similar

**DOI:** 10.1186/1471-2105-10-183

**Published:** 2009-06-15

**Authors:** Karin Verspoor, K Bretonnel Cohen, Lawrence Hunter

**Affiliations:** 1 Center for Computational Pharmacology, University of Colorado Denver School of Medicine, PO Box 6511, MS 8303, Aurora, CO 80045, USA

## Abstract

**Background:**

Recent years have seen an increased amount of natural language processing (NLP) work on full text biomedical journal publications. Much of this work is done with Open Access journal articles. Such work assumes that Open Access articles are representative of biomedical publications in general and that methods developed for analysis of Open Access full text publications will generalize to the biomedical literature as a whole. If this assumption is wrong, the cost to the community will be large, including not just wasted resources, but also flawed science. This paper examines that assumption.

**Results:**

We collected two sets of documents, one consisting only of Open Access publications and the other consisting only of traditional journal publications. We examined them for differences in surface linguistic structures that have obvious consequences for the ease or difficulty of natural language processing and for differences in semantic content as reflected in lexical items. Regarding surface linguistic structures, we examined the incidence of conjunctions, negation, passives, and pronominal anaphora, and found that the two collections did not differ. We also examined the distribution of sentence lengths and found that both collections were characterized by the same mode. Regarding lexical items, we found that the Kullback-Leibler divergence between the two collections was low, and was lower than the divergence between either collection and a reference corpus. Where small differences did exist, log likelihood analysis showed that they were primarily in the area of formatting and in specific named entities.

**Conclusion:**

We did not find structural or semantic differences between the Open Access and traditional journal collections.

## Background

For much of the modern period of biomedical natural language processing (BioNLP) research, work in text mining has focused on abstracts of journal articles. Free and widely available via PubMed/MEDLINE in numbers previously unseen in most statistical text mining work, abstracts enabled a mass of work that has grown remarkably quickly [1]. In recent years, however, there has been both a growing awareness that full text articles are important, and an increasing amount of work using the full text of articles. As early as 2001, Blaschke and Valencia examined recoverability of databased protein-protein interactions from text and concluded that the ability to handle full text would be essential to achieving high-coverage performance [2]. Shah et al. examined the location of biologically relevant words in journal articles and found that although the density of biologically relevant terms is higher in the abstract than in the body of the article, there is much more relevant information in the body of the article than in the abstract [3]. Corney et al. (2004) provided a careful quantification of the costs of failing to work with full text, finding that more than half of the information in molecular biology papers was in the body of the text and not in the abstract [4].

At the same time, it became clear very early on that full text poses challenges that are different from those of abstracts. For example, Tanabe and Wilbur (2002) found that some sections (particularly Materials and Methods) tend to produce much higher rates of false positives on information extraction tasks than others [5]. Furthermore, the substantial length of full text articles as compared to abstracts means that it is likely more difficult to identify individual entities or events, due to the increased linguistic complexity of the text, and the use of longer-distance references. Preprocessing requirements alone can be prohibitively time-costly with full text. Even issues of character encodings and how various journals deal with them – solutions range from inserted gifs to HTML character entities to Unicode – are sufficient to throw off character-offset-based systems, which are increasingly popular.

These problems notwithstanding, recent years have seen an increased emphasis on working with full text papers (see e.g. [6] and [7] for papers that review a substantial amount of work using full text). However, much of this work is done with Open Access journal articles, and with the availability of the PubMed Central Open Access subset [8] of close to 90K biomedical publications (and growing), we expect research on full text to further concentrate on Open Access publications. Such work will assume that the Open Access articles are representative of biomedical publications in general and that methods developed for analysis of Open Access full text publications will generalize to the biomedical literature as a whole. This assumption requires investigation due to the possibility that there exist significant differences in format or content. For instance, the majority of open access journals have to date been exclusively electronic publications, often without formal restrictions on article length (such as the BioMed Central journals), where the lack of strict space constraints could certainly impact the language authors use to present their findings. Furthermore there is at least a perception that these journals often have quicker turnaround on the time from submission to publication [9], and that open access publications have higher community impact [10], both of which could affect the sort of research results that are submitted to open access journals. Similarly, the cost of publication of open access articles may mean that authors tend to submit longer articles combining more research results. The effect of such differences on the textual characteristics of the publications has not to our knowledge been previously explored.

If the basic assumption of the representativeness of Open Access publications is wrong, the cost to the community will be large, including not just wasted resources but also flawed science. This paper sets out to examine that assumption. Our null hypothesis is that traditional and Open Access publications are the same; we seek to find differences between them.

## Results and Discussion

### Results

#### Text collections

We developed or assembled four text collections for comparison.

• **CRAFT **is the Colorado Rich Annotation of Full Text corpus. This is a true corpus in the linguistic sense of that word – a static set of documents with associated linguistic and semantic annotations. The document set was assembled from the PubMed Central Open Access subset [8] with input from the Mouse Genome Informatics group at the Jackson Laboratory to ensure biological relevance. It focuses on mouse genomics. The corpus comprises 97 open access articles containing nearly 750K words.

• **TraJour **(**Tra**ditional **Jour**nals corpus) is a document collection that we assembled from traditional subscription-based journals, with the intent of collecting a set of texts that topically parallels the CRAFT corpus as closely as possible. This parallelism was achieved via shared Gene Ontology annotations (see the Methods section). TraJour consists of 99 articles and almost 600K words.

• **Reference **is a corpus based on the the Wall Street Journal corpus. This is a collection of newspaper articles that has been extensively annotated in the course of the Penn Treebank [11] and PropBank [12] projects. We took the raw text version from the Penn Treebank distribution. It contains about 1.1 million words.

• **BioReference **is a document collection which aims to be representative of full text biomedical publications in general, rather than being tailored to mouse genomics. It was constructed from a random subsample of two document collections: the TREC Genomics Corpus [13], containing full text publications from primarily subscription-based traditional journals, and the PubMed Central Open Access subset, containing exclusively Open Access publications. It is comparable in size to CRAFT and TraJour, at 650K words in 163 articles.

#### Characteristics that we compared in the corpora

We compared the corpora according to various surface-level characteristics as well as several linguistic phenomena. We performed comparisons of the statistical properties of the vocabularies of the corpora in order to identify important variations of language use among them. The two corpora of primary interest are the two semantically comparable corpora – CRAFT, our open access publication corpus, and TraJour, our traditional journal corpus.

We examined the incidence of a number of morphosyntactic/semantic phenomena in the four sets of documents. We selected them because each is known to have consequences for natural language processing: in particular, all of the morphosyntactic phenomena that we examined make the text mining task more difficult by introducing complexity and variability in the linguistic structures found in the text. The linguistic phenomena that we examined were negation, passivization, conjunction, and pronominal anaphora.

To examine negation, we counted every instance of the words *no, not, neither*, and *nor*, as well as the affix *n't*. To examine passivization, we counted instances of the strings *ed by*, *en by*, and *ound by*. This clearly underestimates the number of passives. For example, conjoined passive verbs, as in *eEF2 kinase is phosphorylated and inhibited by SAPK4/p38 delta *[14], will be undercounted. Similarly, intervening adverbials, as in *MAPK is activated primarily by FGF in this context *[15], will cause undercounting, as will bare passives (i.e. those without a subsequent *by*-phrase indicating the agent). However, it yields a reasonable approximation of the number of passives, and the undercounting applies proportionally to all four document sets, so the intra-corpus comparison probably remains valid, although we would need to do a separate analysis to verify this. To examine conjunction, we counted every instance of *and*, *or*, and *but not*. Finally, to examine pronominal anaphora, we counted every instance of any pronoun. In each case, we normalized the counts by the number of words in the corpus.

Table 1 reports the ratio of each phenomenon to the number of words in the four corpora, along with the absolute counts of each. The ratios for the two semantically matched corpora CRAFT (Open Access) and TraJour are similar to each other, and are more similar to each other than they are to the general Reference corpus. When compared to the BioReference corpus, the CRAFT and TraJour corpora are more similar to each other than to the BioReference on the proportion of pronouns and passives in the text. On the proportion of coordination and negatives, the BioReference corpus numbers are about halfway between the CRAFT and TraJour values, though all differences are small. The proximity to the BioReference measures on all of the linguistic dimensions indicates that the differences among them are minor and likely within the range of normal variation for the biomedical literature.

**Table 1:** Incidence of syntactic/semantic phenomena

	CRAFT	TraJour	Reference	BioReference
Document count	97	99	2,500	163

Sentence count	43,694	35,997	53,107	32,895

Avg. Sentence count	450	364	21	202

Token count	717,166	598,331	1,096,976	654,493

Type count	41,574	49,394	40,139	38,801

Stopword count	238,542	193,905	453,264	238,077

Stopword %	33.3%	32.4%	41.3%	36.4%

Avg. Document length	7,393	6,044	439	4,015

Avg. Sentence length	22.5	24.7	26.4	27.8

Types/Tokens	5.8%	8.3%	3.7%	5.9%

Tokens/Types	17.3	12.1	27.3	16.9

Negatives	3,273	2,587	7,605	2,961

Negatives %	0.46%	0.43%	0.69%	0.45%

Coordination	25,237	23,706	26,019	25,059

Coordination %	3.52%	3.96%	2.37%	3.83%

Pronouns	18,874	15,603	57,406	20,699

Pronouns %	2.63%	2.61%	5.23%	3.16%

Passives	2,783	2,587	2,661	3,172

Passives %	0.39%	0.43%	0.24%	0.48%

This table represents the counts of linguistic phenomena determined from our four document sets, CRAFT (open access), TraJour (traditional journals), Reference (Wall Street Journal), and BioReference (full text biomedical publications).

The directions of the differences with the reference corpus are mostly not surprising. Passives are more common in the two semantically matched corpora (0.39% and 0.43%) and in the BioReference (0.48%) than they are in the Reference corpus (0.24%). This accords with the observation that passives are almost caricatural of scientific writing and are quite common in biomedical language [16].

Conjunctions are more frequent in the scientific corpora than in the reference corpus. As Biber et al. [17] point out in their corpus-based study of the grammar of English, comparison of competing hypotheses is a dominant theme in scientific writing. Comparison is often realized by use of conjunctions and by asserting the competing hypotheses. Thus the results are in line with previous research in this area, although a separate analysis would be required to establish what proportion of the conjunctions link competing hypotheses.

The pattern of incidence of negations is also in line with other contrastive reports of negation in the academic and news registers [17]. Incidence of negatives in the two semantically matched corpora and the BioReference reference collection were quite similar – 0.46% for CRAFT, 0.43% for TraJour, and 0.45% for BioReference. However, they were much more common in the WSJ reference than in the three scientific corpora, at 0.69%. This is thought to be related to the use of other terms to express contrast in academic discourse, such as *although, however, nevertheless*, and *on the other hand *[17](81–82).

**Figure 1:**
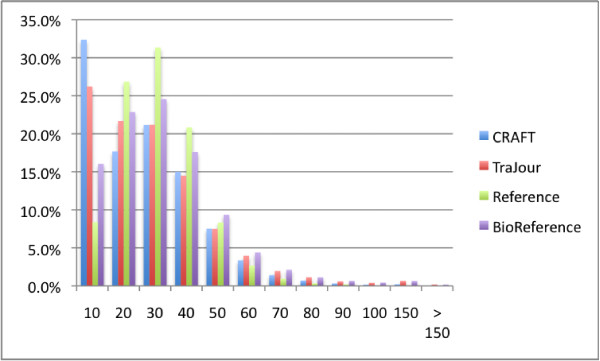
**Sentence length distribution**. Sentence length distributions for the four document sets, measured as the relative proportion of the sentences in the corpus of a particular length. The data here is binned – "10" means a sentence length of 1–10 tokens, "20" 11–20 tokens, etc.

We measured the distribution of sentence lengths because sentence length has implications for syntactic parser performance. Parser accuracy falls as sentence length increases: thus, if there were a difference in sentence lengths between the CRAFT and TraJour corpora, that would indicate that one would present more challenges than the other for an important class of linguistic analysis. Figure 1 shows the histogram of sentence lengths in the four corpora. The mode for both CRAFT and TraJour is at the 0–10 words bin: they do not differ with respect to sentence lengths. In contrast, the WSJ reference differs markedly with respect to sentence length, showing a mode of 20–30 words. Surprisingly, the BioReference also has a mode of 20–30 words; we do not know why it should be more like the WSJ than like the other scientific documents.

The preceding measures are all concerned with linguistic (conjunction, passivization, etc.) or structural (sentence length) feature distributions and their implications for processing difficulty. We now turn to measures that are more reflective of the semantic content of the corpora.

To further explore the possibility of important differences between CRAFT and TraJour, we looked at two measures of lexical difference and similarity. The first of these is Kullback-Leibler divergence [18], or relative entropy, and the second is log likelihood [19].

Kullback-Leibler divergence measures the divergence between two probability distributions. Here, we consider the probability of each word *w *in the vocabulary *V *formed by combining the sets of unique words in two corpora *c*_1 _and *c*_2_. It is calculated as shown in equation (1), and it is converted to a symmetric distance with equation (2).

(1)

(2)

Intuitively, as two distributions become more different, the value for KL divergence increases. We assume a threshold value of 0.005 corresponds to near identity of the distributions. We calculated the KL divergence between CRAFT and TraJour and between each of the two and the reference corpora. We ordered words by frequency in the merged vocabulary of the corpora and then calculated the KL divergence for different values of the top *n *most frequent words, from the 100 most frequent words to the 10,000 most frequent words, comparing the probability distributions for those selected words in the two corpora. We employed Laplace (add-one) smoothing to accommodate for words which occurred in one corpus but not in the other.

**Figure 2:**
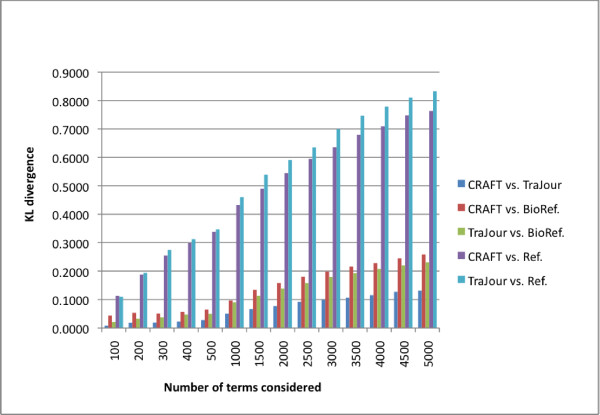
**Kullback-Leibler divergences**. KL divergences at the top *n *terms for CRAFT (open access) versus TraJour (traditional journal) and for each target corpus against the Wall Street Journal reference corpus and the BioReference corpus.

Figure 2 shows the pattern of values; Table 2 shows actual values for a subset of the data points at the two extremes of the frequency list. For the top 500 words, CRAFT and TraJour are nearly identical. In fact we see that the KL-divergence numbers dip below zero in this case. KL-divergence has a theoretical lower bound of 0; the violation of the bound here is a result of error introduced by our smoothing method. This indicates that the two probability distributions have near-complete overlap in the vocabulary for the most frequent terms, and that the probabilities of the shared terms do not differ significantly in the two corpora. The probability distributions for CRAFT and TraJour do not differ above the assumed identity threshold of 0.005 until 500 words are considered, and then only slightly.

**Table 2:** KL divergence of term probability distributions, CRAFT versus TraJour

*n *terms	CRAFT v. TraJour	CRAFT v. BioRef.	TraJour v. BioRef.	CRAFT v. Ref.	TraJour v. Ref.
100	-0.006925696	0.043712192	0.020944793	0.161024124	0.16794174

200	-0.007124725	0.053331913	0.03214335	0.236587232	0.257485466

300	-0.005614059	0.050666423	0.037185528	0.319120526	0.341360939

400	-0.001556702	0.05700178	0.047472912	0.36994002	0.386699809

500	0.007515454	0.064545725	0.04958329	0.411526361	0.421816134

1000	0.041726207	0.096664283	0.089761915	0.513431974	0.548467754

1500	0.06325848	0.134310701	0.11321715	0.577868266	0.641503517

2000	0.078438422	0.158005507	0.138857184	0.642507317	0.69333303

2500	0.098753882	0.180169586	0.157642056	0.697711222	0.746388986

3000	0.108449436	0.19872906	0.179409293	0.746911394	0.817412333

3500	0.118474793	0.215904498	0.193018939	0.794260113	0.87476207

4000	0.132179627	0.228193197	0.207559096	0.830437495	0.904734502

4500	0.145510397	0.244716631	0.21989223	0.872842604	0.942379721

5000	0.152931092	0.258427849	0.230542781	0.89245553	0.969431637

This table shows the KL divergence of the probability distributions of words in the corpora. Each row in the table corresponds to the figure for the top *n *most frequent terms in the corpora.

In contrast, if either corpus is compared against the reference corpus, they are drastically different, with KL divergences for the top 100 words of 0.161 and 0.167, respectively – far above the assumed identity threshold. Even compared with the BioReference corpus, the divergence is well above this threshold (0.044 and 0.021 @100 words), suggesting that there are significant lexical differences between the mouse genome corpora and general biomedical text, while there do not appear to be lexical differences simply due to the mode of publication of the text.

KL divergence scores indicate that CRAFT and TraJour differ very little with respect to semantic content; analysis of the log likelihood scores helps us understand where precisely the two scientific corpora do differ. It will be seen that much of the difference between them is due to formatting and to named entities. Log likelihood values uncover terms that distinguish one corpus from another, by identifying terms that have the most significant relative frequency difference [20]. For each term in the frequency lists derived from two corpora being compared, we calculate the log likelihood statistic. It is based on the expected value for a term *t *in corpus *i*, where *N*_*i *_is the number of word types in corpus *i *and *O*_*i *_is the number of occurrences of *t *in corpus *i*. It is calculated as shown in equations (3)–(4), with (3) representing the expected value for a term in corpus *i*, and (4) the log likelihood for that term. *E*_*i *_tells us how many instances of the term we would expect to see in corpus *i *if the occurrences were evenly distributed across the two corpora. The Log Likelihood measures how far off from that ideal the actual occurrences are. This measure is argued by [19] to be preferable for corpus analysis to statistics that assume a normal distribution (such as the chi squared statistic), due to its ability to more accurately analyze rare events.

(3)

(4)

We see the results of the log likelihood analysis in Tables 3, 4, 5, 6 and 7.

**Table 3:** Log Likelihood analysis of terms in CRAFT vs. TraJour

CRAFT	TraJour	LL
figure		2318.9

doi		1099.3

	window	854.6

	fig	756.7

	text	743.6

	abstract	721.9

mice		678.2

	pp	608.5

hair		601.8

	pdf	588.1

	x1	570.6

	full	550.8

pgc		516.9

?m		502.3

e2		465.6

	chm	460.5

	gp	435.8

ephrin		418.1

qtl		381.5

	view	363.9

°c		338.5

sam68		328.8

atrx		322.0

	bhlh	320.2

ptds		311.8

	version	305.1

olfactory		301.0

	ca	294.9

	mena	294.3

ap		292.2

rb		292.0

sox1		288.2

null		287.4

file		278.4

	p300	270.1

	-catenin	264.0

-1?		262.1

	kinase	256.8

	binding	256.2

	nk	256.0

snail		256.0

-1??		253.6

ited		251.2

	larger	247.3

states		244.0

5?		243.9

	nxt1	241.7

strains		240.3

	articles	239.6

wk		239.4

These are the results of log likelihood analysis of all terms in the CRAFT (open access publications) and TraJour (traditional journals) corpora, ranked by the largest difference.

**Table 4:** Log Likelihood analysis of terms in CRAFT vs. BioReference

CRAFT	BioReference	LL
mice		3755.8

	abstract	1830.7

doi		1650.5

mouse		1489.8

	window	1229.4

	free	1183.7

embryos		1151.8

figure		1017.2

null		922.5

embryonic		657.2

hair		611.3

pgc		539.9

?m		536.9

e2		532.4

olfactory		512.4

ephrin		503.2

development		492.1

mutant		480.3

	view	471.3

wild		465.8

allele		455.9

expression		451.1

qtl		430.6

	version	424.9

gene		416.4

type		411.4

homozygous		407.4

	larger	405.2

knockout		394.6

shh		387.8

heterozygous		384.8

differentiation		376.9

	fig	370.8

°c		361.8

atrx		356.7

sam68		351.5

sections		341.2

	new	336.1

ptds		333.3

ap		332.3

es		324.5

	women	322.5

sox1		320.3

targeted		317.0

annexin		316.8

defects		312.0

limb		311.4

targeting		310.0

	cleavage	306.5

a7		298.7

These are the results of log likelihood analysis of all terms in the CRAFT and BioReference corpora, ranked by the largest difference.

**Table 5:** Log Likelihood analysis of terms in TraJour vs. BioReference

TraJour	BioReference	LL
mouse		1260.6

mice		1219.8

	free	911.8

embryos		704.9

pdf		690.2

text		688.1

pp		569.5

full		543.5

expression		514.6

	medline	512.5

	crossref	497.6

embryonic		479.8

development		447.7

chm		443.4

	patients	425.4

x1		422.4

	risk	372.3

bhlh		357.7

gp		356.8

slap-2		354.7

	figure	354.6

dpc		331.1

jmj		331.1

	women	329.3

tap		326.3

pb		310.7

nxt1		304.5

	isi	297.6

p300		295.8

mena		286.7

endoderm		285.6

hybridization		275.7

	exercise	273.5

cited4		273.4

tbx2		270.5

zfp-57		266.0

otx2		264.6

neural		263.8

	orderarticleviainfotrieve	261.3

	sti	258.9

	abstract	258.6

ko		258.4

mznf8		257.2

heterozygous		255.9

embryo		252.2

gl		249.8

domain		249.5

-catenin		246.6

mutants		245.3

chl1		243.9

These are the results of log likelihood analysis of all terms in the TraJour and BioReference corpora, ranked by the largest difference.

**Table 6:** Log Likelihood analysis of terms in CRAFT vs. Reference

CRAFT	Reference	LL
mice		9705.6

	's	9351.0

	said	7898.0

cells		6565.1

	million	5684.6

expression		5272.3

figure		4528.4

	't	4392.8

	he	4284.3

cell		4224.9

mouse		3914.6

	mr	3850.0

	year	3788.4

gene		3766.7

	company	3362.6

protein		3221.6

	it	3199.2

	to	2986.3

	will	2948.4

type		2833.5

were		2803.0

embryos		2619.0

	its	2564.8

	stock	2475.9

genes		2442.7

doi		2431.4

mutant		2383.2

wild		2317.2

	about	2192.3

	new	2158.6

analysis		2140.2

	his	2107.7

and		1972.7

	who	1843.6

	corp	1769.0

	they	1696.4

null		1689.1

dna		1596.9

in		1585.8

al		1557.6

et		1484.3

	shares	1477.7

	inc	1475.9

	would	1468.6

receptor		1458.6

shown		1396.3

differentiation		1366.1

using		1333.2

	has	1332.9

fig		1326.0

These are the results of log likelihood analysis of all terms in the CRAFT and the general Reference corpora, ranked by the largest difference.

**Table 7:** Log Likelihood analysis of terms in TraJour vs. Reference

TraJour	Reference	LL
	's	8358.8

cells		7680.7

	said	6854.5

expression		5262.0

	million	4975.7

mice		4833.1

	mr	4607.9

cell		4285.6

protein		4074.2

fig		3881.0

	't	3808.8

	he	3660.9

	to	3464.4

mouse		3425.1

	year	3165.5

and		3144.6

	it	2864.9

	will	2858.5

	company	2820.1

et		2690.1

were		2680.4

al		2555.7

gene		2389.7

	stock	2196.0

biol		2180.8

proteins		2163.8

binding		2009.0

type		1990.1

	its	1900.2

domain		1897.9

shown		1873.2

	about	1859.1

embryos		1822.6

	his	1701.0

	they	1700.4

	who	1694.5

	would	1623.4

mutant		1598.6

analysis		1592.6

wild		1591.2

abstract		1560.2

	corp	1546.0

receptor		1537.5

	up	1483.3

activity		1404.0

in		1385.2

expressed		1375.6

genes		1337.5

pp		1316.4

	on	1304.3

These are the results of log likelihood analysis of all terms in the TraJour and the general Reference corpora, ranked by the largest difference.

We can analyze this data in terms of two characteristics: the magnitude of the differences, and the semantic nature of the words in terms of which the various pairs of corpora differ.

With respect to the magnitude of differences, we see that the most different words in the two content-matched corpora, CRAFT and TraJour, are far less different than the most different words between either of those corpora and either of the reference corpora: the most different word between CRAFT and TraJour is *figure*, with a log likelihood of 2318.9, while the most different word between CRAFT and BioReference is *mice *with a log likelihood of 3755.8. The most different word between TraJour and BioReference is *mouse*, with a log likelihood of 1260.6. (The differences between the two content-matched corpora and the WSJ reference corpus are considerably higher, but we omit them from consideration here because the comparison against the BioReference corpus is a much more stringent comparison.) With respect to the semantic content of the words in terms of which the various pairs of corpora differ, we see clear patterns. The six most different words between the two semantically matched corpora CRAFT and TraJour all reflect formatting: *figure *and *doi*, which are overrepresented in CRAFT as compared to TraJour, and *window, fig, text*, and *abstract*, which are overrepresented in TraJour. In fact, of the 50 most different terms between the two corpora, at least a quarter of them reflect formatting differences and artifacts of the text conversion routines – the preceding six terms, plus *pp, ?m, °c, null, -1?, -1??*, and *5?*. Many of the remaining differences are due to the specific named entities that occur in each corpus. However, when we compare either of the two semantically matched corpora CRAFT and TraJour against BioReference, we see content words such as *mice, mouse*, and *embryos *ranked much higher, and we see more overlap among the most significant terms. In Table 8 the top 50 terms, by TF*IDF (Term Frequency * Inverse Document Frequency) calculated with respect to the Reference corpus term document frequencies, are shown and the significant overlap in the vocabularies of CRAFT and TraJour is clear. This indicates that not only are the Open Access and traditional documents similar in terms of surface linguistic phenomena, but that authors talk about the same things in them (in this case, mouse genomics), as compared against a set of documents selected from across all of biomedicine.

**Table 8:** TF*IDF-ranked terms in the corpora

CRAFT		TraJour		Reference		BioReference	
**mice**	0.435821989	**cells**	0.336961638	mr	0.121256579	**cells**	0.320612568
**cells**	0.270086285	**mice**	0.23486562	says	0.118389148	**fig**	0.205308437
**expression**	0.216037704	**expression**	0.23159711	that	0.118092658	cell	0.20214811
**mouse**	0.178144406	**fig**	0.220320662	he	0.102566064	abstract	0.190193446
**cell**	0.172290914	**protein**	0.195781243	market	0.091669921	medline	0.188483175
**gene**	0.163204203	**cell**	0.187501368	's	0.088505453	**protein**	0.177454065
**embryos**	0.151251462	**mouse**	0.167860719	million	0.08479812	fulltext	0.140623811
**protein**	0.14510293	**biol**	0.135330482	is	0.083961293	**expression**	0.138198756
figure	0.12789903	**et**	0.120455574	as	0.081560405	orderarticle...	0.119943839
doi	0.122095859	**gene**	0.117032939	his	0.081293607	**genes**	0.109091523
**genes**	0.120878869	**al**	0.1166477	on	0.079143554	**proteins**	0.106029999
**mutant**	0.119701823	**embryos**	0.113119754	stock	0.078988492	**gene**	0.098232094
null	0.097585044	**proteins**	0.110513285	they	0.078407133	**were**	0.096137225
**type**	0.093527187	domain	0.093587827	at	0.075765418	binding	0.094229295
**wild**	0.085050648	binding	0.093519941	but	0.075548004	window	0.085695981
**differentiation**	0.078946407	**mutant**	0.086691778	billion	0.073818895	induced	0.085566187
**analysis**	0.076546987	**receptor**	0.085026763	have	0.073662149	**biol**	0.085231028
**receptor**	0.075227992	pp	0.081740644	are	0.072364352	ml	0.083458459
**dna**	0.073316012	**mutants**	0.077608416	be	0.071025302	min	0.083015317
**pcr**	0.073162003	abstract	0.077359299	with	0.068584195	**al**	0.078391977
**biol**	0.072197936	**antibody**	0.076735615	it	0.067830211	**et**	0.077346227
**fig**	0.070585942	cdna	0.076317095	was	0.067707989	**analysis**	0.076920249
**were**	0.070224935	**genes**	0.07615871	't	0.066475036	mm	0.073266187
allele	0.069948445	membrane	0.075929698	in	0.065890951	**mice**	0.072205176
**al**	0.067582502	**transcription**	0.073863584	trading	0.065657748	**shown**	0.070980579
**mutants**	0.066734887	**type**	0.073860554	would	0.06509097	data	0.06877046
**embryonic**	0.0644353	**were**	0.072302222	said	0.064915624	ph	0.067550112
**et**	0.06344436	**sequence**	0.070485632	to	0.064419151	activation	0.06720991
staining	0.061271839	kinase	0.070118752	has	0.064175458	**receptor**	0.066788269
neurons	0.059343704	**pcr**	0.070118752	by	0.063766297	**sequence**	0.066026426
**proteins**	0.058555579	**shown**	0.069422034	shares	0.063615252	**antibody**	0.065025557
mm	0.057094213	X1	0.068956563	company	0.063043995	human	0.064973329
olfactory	0.056987095	activation	0.065599127	their	0.062731731	using	0.064071093
**transcription**	0.056130146	**wild**	0.065140388	for	0.062641744	**dna**	0.063146568
**signaling**	0.055582376	**analysis**	0.06314115	bonds	0.061745073	crossref	0.062926201
phenotype	0.052916588	wt	0.062499956	will	0.061422042	activity	0.058794757
observed	0.05206838	**dna**	0.060635915	year	0.061329696	rna	0.058294133
e2	0.051952521	chem	0.060606544	new	0.060716109	observed	0.05785548
**shown**	0.050532729	pdf	0.060433841	**were**	0.06062604	with	0.057545637
homozygous	0.050131504	mrna	0.060175577	or	0.060257745	these	0.057379774
function	0.049871842	rna	0.059552487	an	0.060255469	study	0.056368432
muscle	0.049628485	ca	0.055251655	from	0.059225401	free	0.056039813
data	0.049494253	**differentiation**	0.055139424	we	0.059174038	mediated	0.055983639
**antibody**	0.048131217	insulin	0.05440163	index	0.059103846	serum	0.05494964
chromosome	0.048033587	activity	0.053267608	some	0.058883875	actin	0.054506498
we	0.047444291	expressed	0.053108054	one	0.058690763	kinase	0.053029357
**sequence**	0.047236181	**embryonic**	0.052726312	more	0.058586253	?c	0.052586215
transgenic	0.046764407	**signaling**	0.052369358	stocks	0.058457121	we	0.051671671
using	0.046658651	molecular	0.052281469	sales	0.058224908	figure	0.051378087
pgc	0.045739642	amino	0.052137849	this	0.05791668	amino	0.050216424

These are the top 50 terms in each corpus, by TF*IDF (Term Frequency * Inverse Document Frequency). Terms highlighted in bold in the CRAFT and TraJour columns indicate terms that are shared among these two corpora within the top 50 terms of each corpus; terms highlighted in bold in the BioReference column are shared among all three corpora in the top 50 terms. There is clearly significant overlap between CRAFT and TraJour in their contentful terms.

## Discussion

In terms of linguistic phenomena such as conjunction, passivization, negation, and pronominal anaphora, the content-matched Open Source and traditional publications do not differ from each other. They also do not differ in terms of sentence length. When compared against reference corpora, they do differ from these more general document sets, indicating that if the Open Source and traditional journals did differ from each other, our methods would have uncovered those differences.

The two target corpora analyzed (CRAFT and TraJour) are both in the molecular biology domain, and more specifically mouse genomics. As such, the results and conclusions, strictly interpreted, apply only to the particular datasets we examined. Based on the analysis of the factors that might lead to textual variation (see Background), it would be conservative to assume that these results generalize to the molecular biomedical literature as a whole. We believe that generalizing these results to the entire biomedical literature, or even all peer reviewed scientific publications, is reasonable, although additional testing may be warranted for areas with substantially different cultures of scientific practice.

## Conclusion

We tried hard to find differences between the CRAFT and TraJour document sets. We mostly failed. Research on Open Access documents applies to traditional, subscription-only journals.

## Methods

### Construction of the TraJour corpus

The document set was selected by collecting the set of Gene Ontology annotations with an evidence code of Traceable Author Statement (see Gene Ontology 2000 for an explanation of evidence codes) from the Mouse Genome Institute's Gene Ontology annotation file [21] for documents in the CRAFT corpus, eliminating two annotations that were overly generic (GO:0005515 "protein amino acid binding" and GO:0005634, "cell nucleus"), and then randomly selecting 100 articles from other articles associated with those Gene Ontology terms. These were all identified as coming from traditional subscription-based journals. One of the selected articles was discarded due to our inability to access the full text of the article. The remaining 99 articles form our corpus, and contain over 650K words. Most of these articles were obtained as full text HTML from the individual publisher's websites, though 14 articles were only available as PDFs. To convert those PDFs to plain text, we used a conversion tool from the USC Information Sciences Institute. The HTML files were (imperfectly) processed to handle character entities and to remove javascript, frames, HTML tags and other non-contentful text prior to the analysis.

### Construction of the BioReference corpus

One hundred PubMed identifiers were selected at random from each of two sources: the 2006 TREC Genomics Corpus [13] and the PubMed Central Open Access subset [8]. These two sources were used because they are the only two large collections of full textpublications that we have access to. The TREC Genomics Corpus was collected originally for the Genomics Track of the Text Retrieval Conference. The 2006 corpus contains over 162K articles from 49 journals, ranging from the *American Journal of Epidemiology *to several *American Journal of Physiology *journals (e.g. *Heart and Circulatory Physiology*), and as such the corpus has quite broad coverage of biomedicine despite the "Genomics" name. Our selection included 41 articles from *The Journal of Biological Chemistry*, 12 from *Blood*, 4 each from *Human Reproduction*, *Human Molecular Genetics*, and the *Journal of Applied Physiology*, and 1–3 each from 20 other journals.

The portion of the BioReference corpus randomly selected from the PubMed Central Open Access included publications from *Nucleic Acids Research *(23 articles), *Environmental Health Perspectives *(9 articles), *Ulster Medical Journal *(4 articles), *BMC Genomics *(4 articles), *Medical History *(4 articles) and 44 other journals contributing 1 or 2 articles each.

Three of the articles selected for the PubMed Central dataset were missing from that set. After selecting the files and pre-processing them to extract the plain text, two files from the TREC Genomics collection were found to be empty. The corpus thus consists of 195 files containing content, 97 from the PubMed Central Open Access dataset and 98 from the TREC Genomics dataset. We then eliminated any files less than 1 kb (1024 bytes) in length, as those did not represent full text files. The remaining 163 files comprise a reference set which can be considered to be a balanced sample of both full text Open Access and traditional journal publications indexed in PubMed, and are not oriented on the topics relevant to mouse genomics on which CRAFT and TraJour are focused.

### Computational methods

All the computations described here were implemented in Java within the UIMA (Unstructured Information Management Architecture) [22,23] framework.

### Statistical methods

We have not performed significance testing of the statistical results provided in this paper as we are mostly interested in the qualitative differences that could impact text mining applications, and minor variations will always exist between any particular document corpora. This is a limitation of the approach.

## Authors' contributions

KV conceived the lexical distribution measures, collected and pre-processed the corpora, and designed and carried out the KL divergence, frequency, and log likelihood experiments. KBC conceived, designed, and carried out the linguistic/syntactic experiments. LH contributed to the design of the experiments. KV, KBC, and LH analyzed the results and wrote the paper.
